# The safety and effectiveness of anesthetic drops for intravitreal drug administration: a meta-analysis

**DOI:** 10.3389/fmed.2026.1778281

**Published:** 2026-04-01

**Authors:** Amro Alhazimi

**Affiliations:** Department of Ophthalmology, College of Medicine, Imam Mohammad Ibn Saud Islamic University (IMSIU), Riyadh, Saudi Arabia

**Keywords:** intravitreal injections, lidocaine, pain, proparacaine, topical anesthesia

## Abstract

**Background:**

Intravitreal injections (IVI) are increasingly performed for the management of retinal diseases, yet procedural discomfort remains a common concern. Topical anesthetic drops are frequently used to mitigate pain, but evidence regarding their safety and efficacy compared with alternative anesthetic modalities, such as gels or subconjunctival injections, remains heterogeneous and limited.

**Objective:**

To systematically evaluate the safety and effectiveness of topical anesthetic drops for intravitreal drug administration, with a focus on patient-reported pain, procedural discomfort, and satisfaction compared with other anesthetic methods.

**Methods:**

A systematic literature search was conducted through PubMed, Scopus, EMBASE, ClinicalTrials.gov, and other databases through October 2025. Eligible studies included clinical studies comparing topical anesthetic drops for IVI with other topical anesthetic agents.

**Results:**

Eight studies comprising 637 eyes were included. Topical anesthetic drops demonstrated analgesic efficacy comparable to anesthetic gels and subconjunctival anesthesia, with no statistically significant differences in post-injection pain (SMD −0.11, 95% CI; −0.27 to 0.05, *p* = 0.18), burning sensation (RR 0.77, 95% CI: 0.56, 1.06, *p* = 0.11), or overall patient satisfaction (MD −0.09, 95% CI −0.30 to 0.13, *p* = 0.43). Subgroup analyses confirmed consistent results across different formulations and delivery methods.

**Conclusion:**

Topical anesthetic drops are a safe, effective, and well-tolerated option for IVI, providing analgesia comparable to gels and subconjunctival anesthesia. Their rapid onset and ease of administration support their use as a first-line anesthetic strategy in routine clinical practice, particularly in high-volume injection settings. Further high-quality randomized trials are warranted to confirm these findings across diverse patient populations and clinical contexts.

## Introduction

Intravitreal injection (IVI) is one of the most frequently performed procedures in vitreoretinal subspecialty (1). Although corticosteroids, antibiotics, and antiviral agents have long been administered intravitreally, the use of IVI has expanded significantly following the advent of anti–vascular endothelial growth factor (anti-VEGF) therapy for neovascular age-related macular degeneration. Current indications for anti-VEGF IVI also include diabetic retinopathy and retinal vascular occlusions (2). Over the past decade, IVI has become the foremost ophthalmic intervention, exhibiting an approximately 11-fold escalation in procedural frequency. In light of an aging population and the advent of novel therapeutic modalities, the prevalence of IVI is projected to continue increasing (3, 4). However, pain during IVI is common, particularly in patients requiring repeated treatments. Local anesthesia reduces discomfort and prevents complications related to pain-induced ocular movements (5, 6). Local anesthesia for intravitreal drug delivery can be achieved through several techniques, including peribulbar and subconjunctival injections, as well as topical anesthetic drops or gel applied to the injection site directly or using soaked cotton-tipped applicators saturated with topical anesthetic shortly before the procedure (7).

Topical anesthetic drops act by blocking cell membrane depolarization, reducing sodium ion permeability, and thereby inhibiting pain transmission in the cornea, conjunctiva, and sclera. The most commonly used agents are proparacaine hydrochloride (HCl), tetracaine HCl, and lidocaine. The anesthetic effect begins within 15–20 s and lasts approximately 15 min (7, 8). However, topical anesthetic drops may cause a burning sensation, and prolonged use can lead to decreased corneal sensitivity, keratitis, corneal opacity, and vision loss. Topical anesthetic gels, including 0.5% tetracaine HCl gel, lidocaine HCl, and preservative-free lidocaine HCl gel, provide prolonged anesthesia as they are less susceptible to dilution by tears. These gel formulations are generally well tolerated. However, their use prior to povidone-iodine application has been associated with weaker antisepsis and higher bacterial survival rate, which may lead to an increased risk of post-injection endophthalmitis (9, 10). On the other hand, some topical anesthetic drops have been shown to have antibacterial effects at clinically used concentrations *in vitro*. This was attributed to bacterial cell membrane disruption causing leakage of cellular components and lysis (11). Furthermore, subconjunctival administration of anesthetic agents frequently results in conjunctival hyperemia at the site of injection and is often associated with localized hemorrhage and/or chemosis, particularly among patients receiving aspirin or other anticoagulant therapy (12, 13).

Multiple anesthetic agents and formulations have been implemented during IVI to alleviate procedure-related patient discomfort. However, existing evidence is largely subjective and heterogeneous, making it difficult to define the most effective anesthetic agent (10). Although topical anesthetic drops are commonly used to enhance patient comfort during IVI, the evidence regarding their safety and effectiveness remains scattered and inconsistently reported. Existing studies vary in design, patient populations, outcome measurements, and anesthetic protocols, making it difficult to draw firm conclusions about their efficacy as a standalone anesthetic option. Many studies are underpowered, rely on subjective pain assessments, and provide limited data on adverse events or procedural complications, highlighting the need for a high-quality, systematic evaluation. This study aims to systematically evaluate the safety and effectiveness of topical anesthetic drops for intravitreal injections, comparing them with anesthetic gels and subconjunctival injections in terms of patient-reported pain, procedural comfort, and complication rates.

## Methodology

### Study methodology

This systematic review was conducted following the Preferred Reporting Items for Systematic Reviews and Meta-Analyses (PRISMA) guidelines (14), and the recommendations of the Cochrane Collaboration. The review protocol was prospectively registered in the PROSPERO database (15).

### Eligibility criteria

This review included all clinical studies evaluating the safety and effectiveness of topical anesthetic drops for intravitreal injections, including comparisons with anesthetic gels or subconjunctival anesthesia. Eligible studies reported outcomes related to patient-reported pain, procedural discomfort, or injection-related complications. Studies lacking relevant outcome data, as well as reviews, animal studies, clinical guidelines, case reports, letters, editorials, conference abstracts, commentaries, and book chapters, were excluded. Two independent reviewers screened titles, abstracts, and full texts to determine eligibility, resolving disagreements through discussion. The selection process and reasons for exclusion were systematically documented using a PRISMA flow diagram. No restrictions were applied regarding patient age, sex, underlying ocular condition, or geographic location.

### Searching strategy

A comprehensive systematic literature search was performed from database inception through 23 October 2025 across multiple electronic sources, including PubMed, Google Scholar, Scopus, SIGLE, Virtual Health Library (VHL), New York Academy of Medicine (NYAM), ClinicalTrials.gov, the metaRegister of Controlled Trials (mRCT), EMBASE, and the World Health Organization International Clinical Trials Registry Platform (WHO ICTRP).

The search strategy was carefully designed to maximize sensitivity and comprehensiveness by combining controlled vocabulary, such as Medical Subject Headings (MeSH), with relevant free-text terms. Key terms included “intravitreal,” “anesthetic,” “anesthetics,” “anesthesia,” “oxybuprocaine,” “lidocaine,” “tetracaine,” and “proparacaine,” which were used in various combinations with Boolean operators (AND, OR) and database-specific truncation or wildcard functions as appropriate.

To ensure thorough coverage, the reference lists of all included studies were manually reviewed to identify additional eligible articles not captured in the initial database search. This iterative cross-referencing process was repeated until no additional relevant studies were identified.

### Data extraction

Data extraction was performed using a structured Microsoft Excel spreadsheet to ensure accuracy and consistency. Study-level characteristics, including title, first author, publication year, design, duration, and geographic location, were recorded, along with methodological details such as study endpoints and follow-up duration. Baseline patient demographics, including sample size, number of treated eyes, age, race, prior intravitreal injections, concomitant medications, and relevant comorbidities, were collected. Details of local anesthesia, including agent, application method, and injection indication, were also retrieved. Extracted outcomes included patient-reported pain, need for additional anesthetic or systemic analgesia, post-injection vitreous reflux, overall satisfaction, and procedure-related adverse events. This systematic approach ensured comprehensive and consistent data capture for analysis.

### Risk of bias and quality assessment

The risk of bias in randomized clinical trials was assessed using the Cochrane Collaboration’s tool in conjunction with ROBVIS (16, 17). This tool assesses five domains: the randomization process, deviations from the intended intervention, missing outcome data, outcome measurement, and reporting of results. The methodological quality of observational studies was appraised using the National Institutes of Health (NIH) quality assessment tool. Studies were categorized as good, fair, or poor based on scores of >65%, 30–65, and <30%, respectively (18).

### Statistical analysis

Continuous outcomes were synthesized using either the standardized mean difference (SMD) or weighted mean difference (WMD). For dichotomous outcomes, pooled estimates were calculated as risk ratios (RR) or odds ratios (OR) with corresponding 95% confidence intervals (CIs). A fixed-effect model was applied when study results were sufficiently homogeneous; otherwise, a random-effects model was employed to account for between-study variability. Statistical heterogeneity was assessed using Higgins’ I^2^ statistic, with values >50% indicating substantial heterogeneity, and the Cochrane Q test (Chi^2^), with significance set at *p* < 0.10 (19). All analyses were conducted using Review Manager version 5.4 (RevMan 5.4) and Comprehensive Meta-Analysis version 3 (CMA V3) software. A *p*-value <0.05 was considered statistically significant (20, 21).

## Results

**Figure 1:**
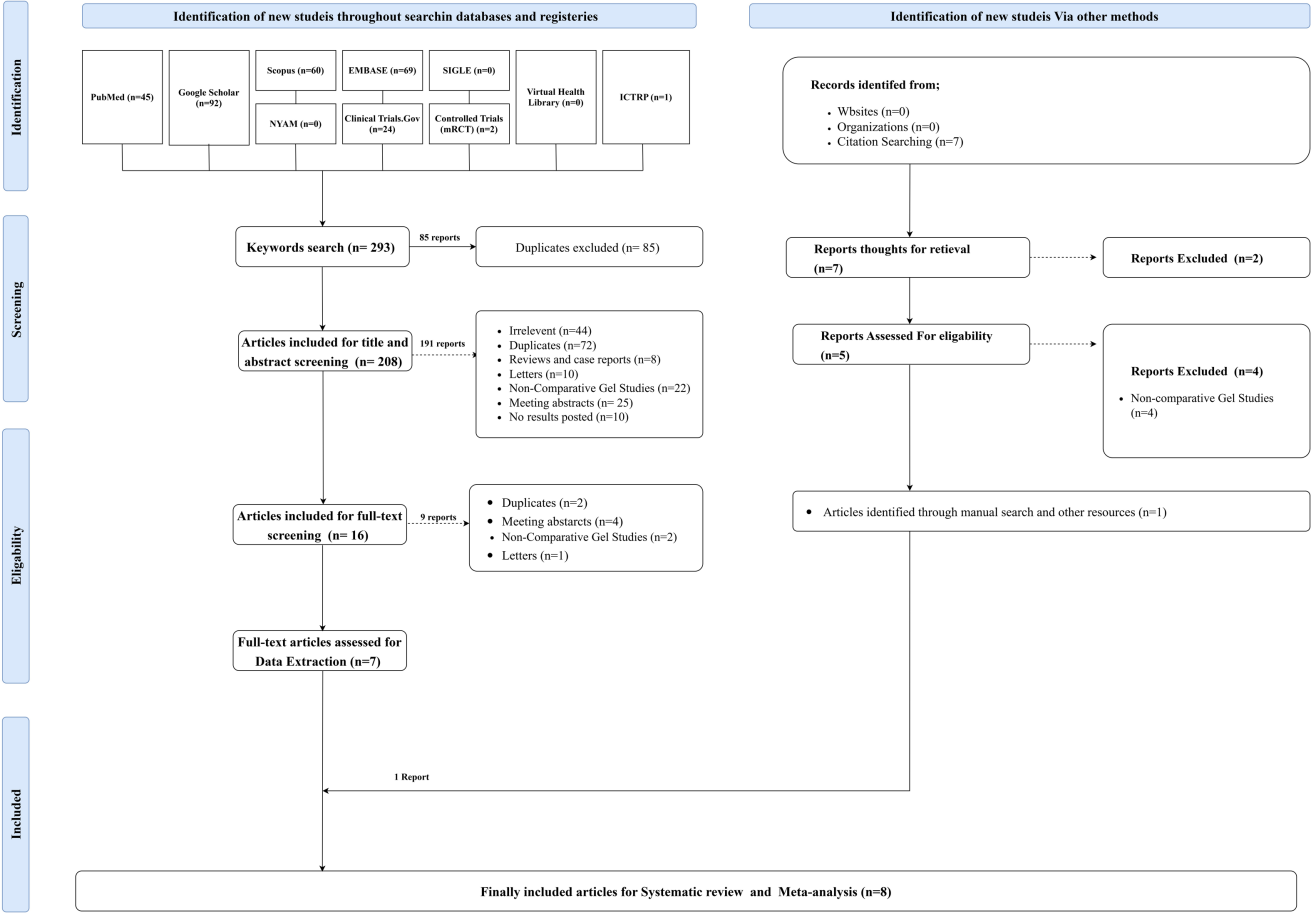
PRISMA flow chart showing the process of the literature search, title, abstract, and full text screening, systematic review, and meta-analysis.

The systematic literature search initially identified 293 articles, of which 85 were duplicates, leaving 208 records for title and abstract screening. Sixteen articles were deemed eligible for full-text review, and seven met the inclusion criteria for data extraction. An additional study was identified through manual searching, resulting in a total of eight studies included in the meta-analysis (Figure 1).

### Baseline demographic characteristics

The present study included eight articles, encompassing 637 eyes. There were four randomized controlled trials, and four prospective observational studies. Proparacaine eye drops were utilized in three studies, while anesthetic gel was employed in six studies (6, 22–28). Of the treated eyes, 317 (49.76%) received topical anesthetic drops for intravitreal drug administration, 258 (40.5%) received anesthetic gel. The mean age of the included patients ranged from 69.4 to 80.3 years. There were 227 (45.5%) females and 272 (54.5%) males. The mean number of previous injections ranged from 9.03 to 18 across the included studies (Table 1).

**Table 1:** Demographic characteristics of the included studies.

Study ID		Region	Study design	Intervention	Control	Timing of anesthesia	Sample size		Age (Years)		Gender				Previous injections		Quality assessment	
											Females		Males					
							Drops	Control	Drops	Control	Drops	Control	Drops	Control	Drops	Control		
							Number	Number	Mean ± SD	Mean ± SD	Number	Number	Number	Number	Mean ± SD	Mean ± SD	%	Decision
1	Alex et al., 2021 (22)	USA	Prospective	0.5% Proparacaine drops	3.5% lidocaine gel	5–7 min	107	109	79.9 ± 11.0	80.3 ± 10.2	51	56	56	53	NR	NR	78.57%	Good
2	Davis et al., 2012 (24)	USA	RCT	Proparacaine drops	3.5% lidocaine gel	~20–70 s before injection	40	40	80.18 ± 8.62	75.60 ± 12.08	22	18	16	24	10.50 ± 10.01	9.03 ± 7.82	___	___
3	de Andrade et al., 2014 (23)	Brazil.	RCT	0.5% Proparacaine eye drops	3.5% lidocaine gel	NR	31	31	NR	NR	17	14	14	17	NR	NR	___	___
4	Gregori et al., 2012 (25)	USA	A Randomized Clinical Trial	4% Liquid Lidocaine	3.5% lidocaine gel	NR	50	50	74 ± 12		2		48		18 ± 15		___	___
5	KADERLI et al., 2006 (26)	Turkey	Prospective	Lidocaine 4%	Subconjunctival anesthesia with lidocaine 4%	5 min before intravitreal injection	28	28	59 (44–71)*		15		13		NR	NR	78.57%	Good
6	Karaba et al., 2015 (27)	Turkey	A Randomized Clinical Trial	Topical proparacaine drops + 4% lidocaine-pledget	subconjunctival lidocaine injection+ 4% lidocaine-pledget	Immediately before intravitreal injection	31	32	63.1 ± 2.1	60.6 ± 2.7	16	16	15	16	NR	NR	___	___
7	POLLACK et al., 2010 (28)	USA	Prospective	0.5% Proparacaine drops	3.5% lidocaine gel	NR	10	10	NR	NR	NR	NR	NR	NR	NR	NR	71.42%	Fair
8	Rifkin et al., 2012 (6)	USA	Prospective	Proparacaine HCl drops	Tetracaine HCl 0.5% gel	Applied 3 times over 5 min	20	20	NR	NR	NR	NR	NR	NR	NR	NR	78.57%	Good

RCT, randomized controlled trials; NR, not reported. *Data reported in the form of median and range.

### Risk of bias and quality assessment

**Figure 2:**
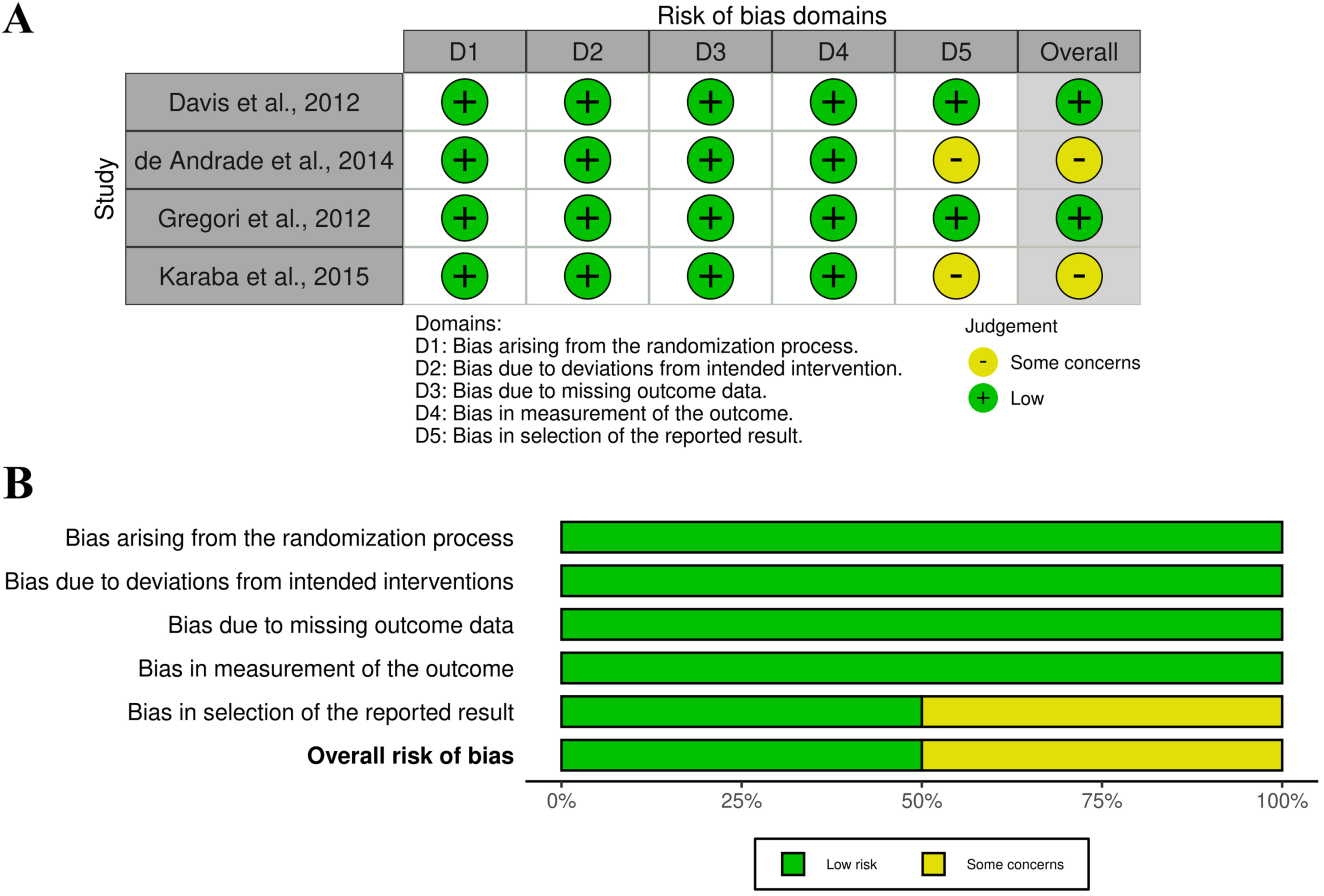
**(A)** Risk of bias graph, **(B)** risk of bias summary: review authors’ judgments about each risk of bias item presented as percentages across all included studies.

All the included observational studies were good quality apart from Pollack et al. (28) which showed fair quality. Overall, the included randomized controlled trials demonstrated a predominantly low risk of bias across most assessed domains. Davis et al. (24) and Gregori et al. (25) were judged to have a low risk of bias in all five domains, resulting in an overall low risk of bias. In contrast, de Andrade et al. (23) and Karaba et al. (27) raised some concerns related to selective reporting (Domain 5), which led to an overall judgment of some concerns, despite low risk of bias in the remaining domains. (Table 1; Figure 2).

### Study endpoints

#### Post-injection pain and discomfort

**Figure 3:**
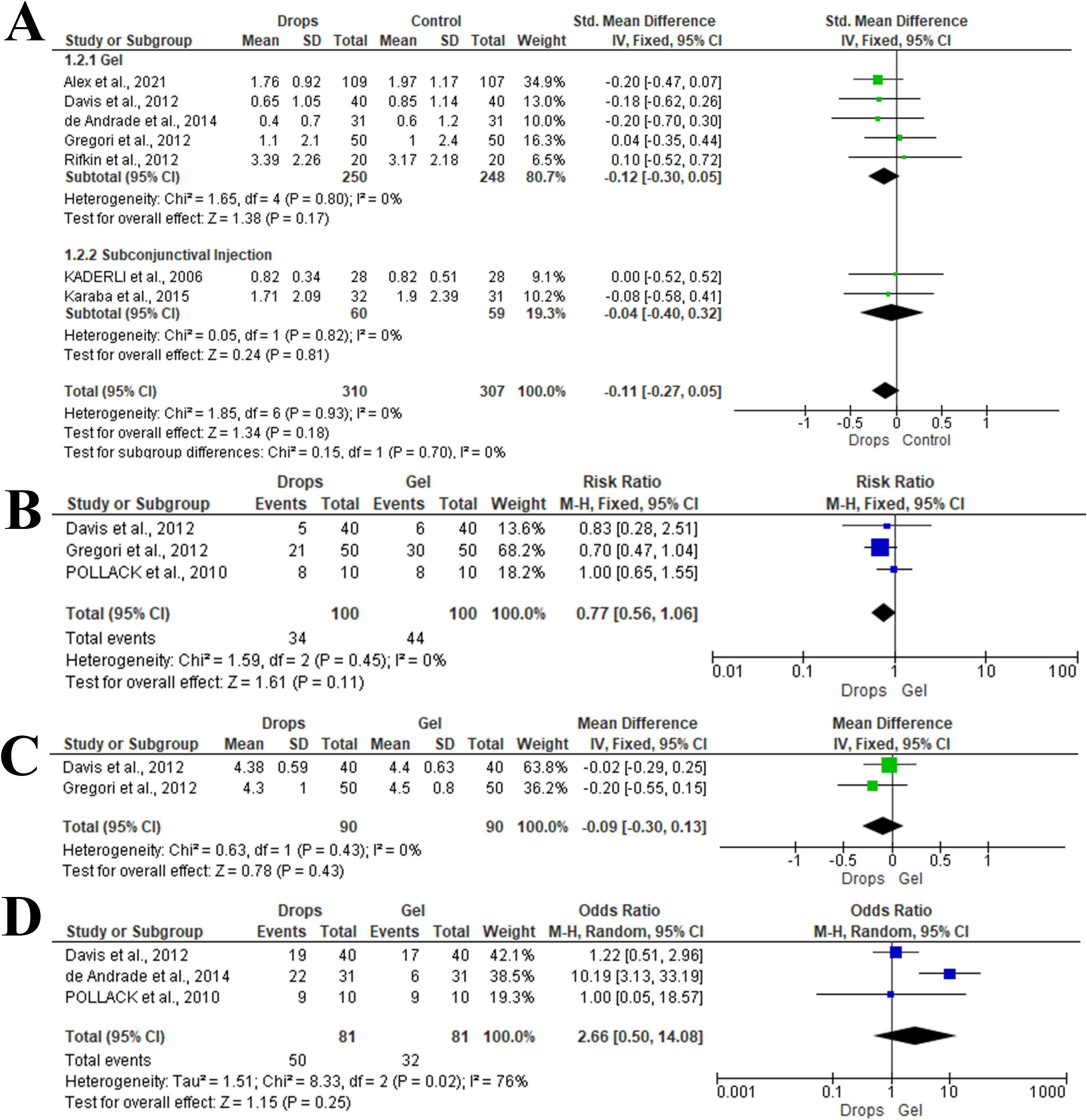
Forest plot of summary analysis of the **(A)** Mean difference and 95% CI of the mean post-injection pain score between topical anesthetic drops and control groups. **(B)** Risk ratio and 95% CI of the burning pain sensation between topical anesthetic drops and anesthetic gel. **(C)** Mean difference and 95% CI of the mean overall satisfaction score between topical anesthetic drops and anesthetic gel. **(D)** Odds ratio and 95% CI of the excellent or very good satisfaction between topical anesthetic drops and anesthetic gel. Size of the blue squares is proportional to the statistical weight of each trial. The grey diamond represents the pooled point estimate. The positioning of both diamonds and squares (along with 95% CIs) beyond the vertical line (unit value) suggests a significant outcome (IV = inverse variance).

Seven articles including 617 eyes reported the mean difference in post-injection pain score between topical anesthetic drops and control groups (6, 22–27). Of the eyes, 250 received topical anesthetic drops compared to 248 who received anesthetic gel, and 60 received topical anesthetic drops compared to 59 who received subconjunctival injections. In the fixed effect model (I^2^ = 0%, *p* = 0.93), there was no statistically significant difference between topical anesthetic drops and control groups (SMD; −0.11, 95%CI; −0.27, 0.05, *p* = 0.18). Subgroup analysis based on the type of control group revealed no statistically significant difference between topical anesthetic drops and anesthetic gel (SMD; −0.12, 95%CI; −0.30, 0.05, *p* = 0.17) and subconjunctival injections (SMD; −0.04, 95%CI; −0.40, 0.32, *p* = 0.81). (Figure 3A).

#### Burning pain

The risk of burning pain sensation after topical anesthetic drops compared to anesthetic gel group was reported within three articles, including 200 eyes (24, 25, 28). The risk of burning pain did not differ significantly between topical anesthetic drops and anesthetic gel (*p* = 0.11) with an RR of 0.77 (95%CI; 0.56, 1.06) in the fixed effect model (I^2^ = 0%, *p* = 0.45). (Figure 3B).

#### Overall satisfaction

Two studies included 180 eyes evaluated the mean difference in overall satisfaction scores between topical anesthetic drops and anesthetic gel groups (24, 25). There was no statistically significant difference between topical anesthetic drops and anesthetic gel groups (MD; −0.09, 95%CI; −0.30, 0.13, *p* = 0.43) in the fixed effect model (I^2^ = 0%, *p* = 0.43). (Figure 3C).

#### Patients excellent/very good satisfaction

The likelihood of excellent or very good satisfaction between topical anesthetic drops and 3.5% anesthetic gel groups was reported within three articles, including 162 eyes (23, 24, 28). Pooling the data in the random-effects model (I^2^ = 76%, *p* = 0.02) revealed lower odds of excellent or very good satisfaction in the topical anesthetic drops group compared with lidocaine gel (OR; 2.66, 95%CI; 0.50, 14.08, *p* = 0.25). (Figure 3D).

## Discussion

Topical anesthetic drops represent the most commonly utilized modality for pain management during intravitreal injections, owing to their rapid onset of action and ease of administration (29). The present meta-analysis demonstrates that topical anesthetic drops provide analgesic efficacy comparable to anesthetic gels and subconjunctival anesthesia, with no statistically significant differences in post-injection pain, burning sensation, or overall patient satisfaction. Their favorable safety profile, combined with widespread availability and practical ease of use, supports the continued utilization of topical anesthetic drops as a reliable first-line strategy for routine intravitreal injection protocols.

Pain control during IVI is a key determinant of patient comfort and procedural acceptance, particularly in individuals requiring repeated treatments. In the present meta-analysis, topical anesthetic drops provided analgesia comparable to anesthetic gels and subconjunctival anesthesia. The absence of heterogeneity across studies suggests consistent effectiveness of topical anesthetic drops across diverse clinical settings and patient populations. Although subconjunctival anesthesia has traditionally been perceived as offering superior analgesia, our findings indicate that this perceived advantage does not confer clinically meaningful improvements in patient-reported pain. In support of these observations, Shiroma et al. (30) reported that pain during intravitreal injections was generally mild across all anesthetic techniques, with no single method demonstrating superiority. Consistent with our findings, Han et al. (7) reported that no single anesthetic agent or method of delivery demonstrated statistically significant superiority over others in terms of analgesic efficacy, and that anesthesia-related adverse events were infrequent and clinically insignificant.

Analgesic efficacy was consistently comparable across these methods, with no statistically significant improvement observed with higher concentrations, gel formulations, or more invasive delivery techniques. These findings indicate that simple topical anesthetic drops provide analgesia equivalent to more complex approaches. This equivalence likely reflects the shared sodium channel-blocking mechanism of these agents and the superficial nature of ocular nociceptive pathways, suggesting that more intensive or invasive anesthetic strategies confer limited additional clinical benefit (31, 32). Topical anesthetic drops demonstrated a favorable safety and tolerability profile across the included studies. The risk of burning sensation following anesthetic gel or drop application was not significantly different, indicating that topical anesthetic drops do not increase procedural discomfort. Similarly, pooled analyses of overall patient satisfaction revealed no significant differences between topical anesthetic drops and comparator modalities, with most patients reporting high levels of procedural comfort.

The findings of this review have several important implications for clinical practice. Given their demonstrated efficacy, safety, and patient acceptability, topical anesthetic drops may be regarded as the first-line option for intravitreal injections. Their rapid onset of action and ease of administration render them particularly advantageous in high-volume injection clinics, where procedural efficiency is critical. Furthermore, the absence of clinically meaningful differences between anesthetic formulations or delivery methods indicates that clinicians can select agents based on availability, cost, or personal preference without compromising patient comfort. More invasive approaches, such as subconjunctival anesthesia, may be reserved for selected cases with heightened pain sensitivity or anxiety; however, current evidence supports the routine use of topical anesthetic drops as both a practical and effective strategy (33). However, the results of this systematic review should be interpreted with caution due to heterogeneity across studies in design, anesthetic agents and formulations, delivery methods, outcome measures, and follow-up duration. Pain and satisfaction were based on subjective patient-reported measures. Variability in assessment tools across studies may have introduced measurement bias and contributed to heterogeneity; therefore, these findings should be interpreted with caution. Although random-effects modeling and subgroup analyses were used to address variability, residual confounding cannot be excluded. Additionally, the limited number of high-quality randomized trials underscores the need for further research to confirm these findings in diverse clinical settings.

## Conclusion

Topical anesthetic drops provide a safe, effective, and well-tolerated option for intravitreal injections, offering adequate pain control with minimal adverse effects. Their analgesic efficacy is comparable to anesthetic gels, and subconjunctival anesthesia, while their ease of administration makes them particularly suitable for routine clinical practice and high-volume injection settings. These findings support the use of topical anesthetic drops as a first-line anesthetic strategy, although further high-quality randomized trials are warranted to confirm these results across diverse patient populations and clinical contexts.
